# Lectures in Bellevue Medical College

**Published:** 1865-10

**Authors:** 


### Lectures in Bellevue Medical College.

Lectures commenced in the Bellevue Hospital Medical College Oct. 11. The vacancy in the Faculty, occasioned by the sudden death of Timothy Childs, has been filled by the transfer of Prof. Smith from that Chair of Surgery to that of Anatomy, while that of the Principles of Surgery have been added to the Chair of Military Surgery, filled by Prof. Hamilton.

